# Coincident Pre-Diabetes Is Associated with Dysregulated Cytokine Responses in Pulmonary Tuberculosis

**DOI:** 10.1371/journal.pone.0112108

**Published:** 2014-11-13

**Authors:** Nathella Pavan Kumar, Vaithilingam V. Banurekha, Dina Nair, Rathinam Sridhar, Hardy Kornfeld, Thomas B. Nutman, Subash Babu

**Affiliations:** 1 National Institutes of Health—International Center for Excellence in Research, Chennai, India; 2 National Institute for Research in Tuberculosis, Chennai, India; 3 Government Stanley Medical Hospital, Chennai, India; 4 Department of Medicine, University of Massachusetts Medical School, Worcester, Massachusetts, United States of America; 5 Laboratory of Parasitic Diseases, National Institute of Allergy and Infectious Diseases, National Institutes of Health, Bethesda, Maryland, United States of America; University of Cape Town, South Africa

## Abstract

**Background:**

Cytokines play an important role in the pathogenesis of pulmonary tuberculosis (PTB) - Type 2 diabetes mellitus co-morbidity. However, the cytokine interactions that characterize PTB coincident with pre-diabetes (PDM) are not known.

**Methods:**

To identify the influence of coincident PDM on cytokine levels in PTB, we examined circulating levels of a panel of cytokines in the plasma of individuals with TB-PDM and compared them with those without PDM (TB-NDM).

**Results:**

TB-PDM is characterized by elevated circulating levels of Type 1 (IFNγ, TNFα and IL-2), Type 17 (IL-17A and IL-17F) and other pro-inflammatory (IL-1β, IFNβ and GM-CSF) cytokines. TB-PDM is also characterized by increased systemic levels of Type 2 (IL-5) and regulatory (IL-10 and TGFβ) cytokines. Moreover, TB antigen stimulated whole blood also showed increased levels of pro-inflammatory (IFNγ, TNFα and IL-1β) cytokines as well. However, the cytokines did not exhibit any significant correlation with HbA1C levels or with bacterial burdens.

**Conclusion:**

Our data reveal that pre-diabetes in PTB individuals is characterized by heightened cytokine responsiveness, indicating that a balanced pro and anti - inflammatory cytokine milieu is a feature of pre-diabetes - TB co-morbidity.

## Introduction

Pre-diabetes (PDM) or intermediate hyperglycemia is a high risk state for diabetes that is defined by glycemic variables that are higher than normal, but lower than diabetic thresholds [1]. The prevalence of PDM is increasing worldwide, and it is estimated that over 470 million people will have PDM by 2030 [2]. PDM is associated with the simultaneous presence of insulin resistance and pancreatic beta-cell dysfunction - abnormalities that start before changes in glycemic control are detectable [1], [3]. Observational evidence shows associations between PDM and early forms of complications known to occur in typical diabetes including nephrophathy, small fiber neuropathy, retinopathy and macrovascular disease [1], [3]. Typically 5–10% of individuals with PDM become diabetic every year, although the conversion rates vary with population characteristics and definition of PDM [4], [5].

Type 2 DM is one of the major risk factors for the development of active pulmonary tuberculosis (PTB) and this interaction is typically characterized by elevated systemic levels of pro-inflammatory cytokines especially Type 1 and Type 17 cytokines [6], [7], cytokines that appear to play an important role in pathogenesis. Whether PDM is also associated with increased susceptibility to active PTB or whether this interaction is associated with a dysregulated cytokine environment is not known. Epidemiological studies have shown a high prevalence of PDM in PTB [8], [9]. In one study from India, a country with extremely high burdens of both TB and DM, it was shown that among newly diagnosed TB patients, 25% had DM while another 25% had PDM, numbers that are in stark contrast to DM and PDM in the general population where the prevalences are 10% and 8%, respectively [8]. Since DM is not only associated with an increased risk of developing active TB but also with TB disease severity and treatment outcomes [10], it stands to reason that PDM might also potentially have an impact on the outcome of PTB.

In this study, we sought to delineate the influence of PDM on the systemic and antigen -specific cytokine response in patients newly diagnosed with active PTB. We focused on a large panel of Type 1, Type 2, Type 17, regulatory and other pro – inflammatory cytokines in plasma and TB antigen-stimulated whole blood from individuals with active TB and coincident PDM (TB-PDM) and compared these to those with active TB but without pre-diabetes (TB-NDM). We show that the pre-diabetic state at the time of PTB causes systemic increases in the levels of most of the pro – and anti – inflammatory cytokines, some appearing to be TB antigen-induced. Thus, our data suggest that heightened cytokine levels in active PTB may be associated with even moderate glycemic control.

## Materials and Methods

### Ethics statement

All individuals were examined as part of a natural history study protocol approved by the Institutional Review Board of the National Institute of Research in Tuberculosis (NCT01154959), and informed written consent was obtained from all participants.

### Study Population

We studied a group of 90 individuals with active PTB attending the TB Clinic at Stanley Medical Hospital, Chennai, India. Patients were prospectively/consecutively enrolled with 48 having PDM and 42 having normal HbA1c levels (NDM). PTB was diagnosed on the basis of sputum smear and culture positivity. Bacterial burdens were assessed by measuring smear grades following Ziehl-Nielsen staining of sputum smears. PDM was diagnosed on the basis of glycated hemoglobin (HbA1c) levels, according to the American Diabetes Association criteria (all PDM individuals had HbA1c levels between 5.7 and 6.4%). All the individuals were HIV seronegative. The two groups did not differ significantly in terms of the sputum smear grade. All individuals were anti-tuberculous treatment naïve. Anthropometric measurements, including height, weight, and waist circumference, and biochemical parameters, including random plasma glucose, fasting lipid profile and HbA1c were obtained using standardized techniques as detailed elsewhere [11].

### ELISA

Plasma cytokines and chemokines were measured using a Bioplex multiplex cytokine assay system (Bio-Rad, Hercules, CA). The parameters analyzed were IFNγ, TNFα, IL-2, IL-17A, IL-4, IL-5, IL-10, IL-6, IL-12 and GM-CSF. Plasma levels of TGFβ, IL-1α, IL-1β (all R& D Systems); IL-17F (Biolegend); IL-22 (eBioscience); Type 1 interferons (IFNs) - IFNα (multiple subtypes) and IFNβ (PBL Interferon Source) were measured by ELISA.

### Quantiferon supernatant ELISA

Whole blood from a subset of TB-PDM and TB-NDM individuals (n = 22 each) was incubated with either no antigen or TB antigen (ESAT-6, CFP-10, TB 7.7) according to the manufacturers instructions using the Quantiferon in Tube Gold kit. The unstimulated or TB antigen stimulated whole blood supernatants were then used to analyze the levels of IFNγ, TNFα, IL-17A and IL-1β using the Duo-set ELISA kits from R& D systems.

### *Ex vivo* analysis

All antibodies used in the study were from BD Biosciences/BD Pharmingen/eBioscience/R&D systems. Whole blood was used for ex vivo phenotyping and it was performed on all 90 individuals. Naïve and memory T cell phenotyping was performed using CD45RA and CCR7 staining on CD4^+^ and CD8^+^ T cells and natural regulatory T cell (nTregs) phenotyping using CD25, Foxp3 and CD127. A representative graph showing the gating strategy for CD4^+^ and CD8^+^ T cell subsets is depicted in Figure S1.

### Statistical Analysis

Geometric means (GM) were used for measurements of central tendency. Statistically significant differences between two groups were analyzed using the nonparametric Mann-Whitney U test followed by Holm's correction for multiple comparisons. Correlations were calculated using Spearman rank correlation. Analyses were performed using GraphPad PRISM Version 5.01.

## Results

### Study population characteristics

The baseline characteristics including demographic and biochemical features of the study population are shown in Table 1. As can be seen, compared to subjects without pre-diabetes (TB-NDM), those with pre-diabetes and TB (TB-PDM) had higher glycated hemoglobin but did not differ significantly in other biochemical parameters including random blood glucose, serum cholesterol, HDL, LDL and triglycerides levels. In addition, the baseline immunological profile reveals significantly lower frequencies of CD4^+^ regulatory T cells (p = 0.0009) and significantly higher frequencies of CD8^+^ effector memory T cells (p = 0.0047) in TB-PDM individuals.

**Table 1:** Demographics, biochemistry and immunology profile of TB-PDM and TB-NDM individuals.

Study Demographics	PTB-PDM	PTB-NDM	P value
Age (yrs)	42.5 (18–65)	39.5 (20–65)	NS
Sex M/F	38/10	36/6	NS
BMI (kg/m^2^)	22.5 (16.4–24.5)	20.4 (14.6–22.3)	NS
Glycated Hemoglobin(%)	5.92 (5.71–6.44)	5.25 (4.04–5.65)	NS
Random glucose (mg/dl)	99 (66–161)	100 (78–156)	NS
Total cholesterol (mg/dl)	168 (142–202)	146 (124–205)	NS
Serum triglycerides (mg/dl)	102 (64–424)	98 (56–384)	NS
High density lipoprotein cholesterol (ml/dl)	37 (29–59)	42 (25–66)	NS
Low density lipoprotein cholesterol (ml/dl)	104 (44–180)	96 (52–146)	NS
**Immunological Parameters**			
CD4^+^ Naïve Cells (CD45RA^+^CCR7^+^)	37.5(10–77)	34(11–67)	NS
CD4^+^ Central Memory Cells (CD45RA-CCR7^+^)	32(11–86)	27.5(15–64)	NS
CD4^+^ Effector Memory Cells (CD45RA-CCR7-)	24.05(10–55)	29.5(9–62)	NS
CD4^+^ Regulatory T Cells (CD25^+^FoxP3^+^CD127^dim^)	3.3015(2.129–7.798)	4.659(1.968–9.295)	p = 0.0009
CD8^+^ Naïve Cells (CD45RA^+^CCR7^+^)	28(18–48)	25(10–69)	NS
CD8^+^ Central Memory Cells (CD45RA-CCR7^+^)	1.75(0.9–7.9)	1.1(0.5–7.1)	p = 0.0047
CD8^+^ Effector Memory Cells (CD45RA-CCR7-)	34.5(10–81)	30(11–79)	NS

The values represent geometric means and range (except for age where median and range are shown). P values were calculated using the Mann-Whitney test (except for sex which was tested by Fishers exact test).

### TB-PDM is associated with increased circulating levels of Type 1 and Type 17 cytokines

**Figure 1:**
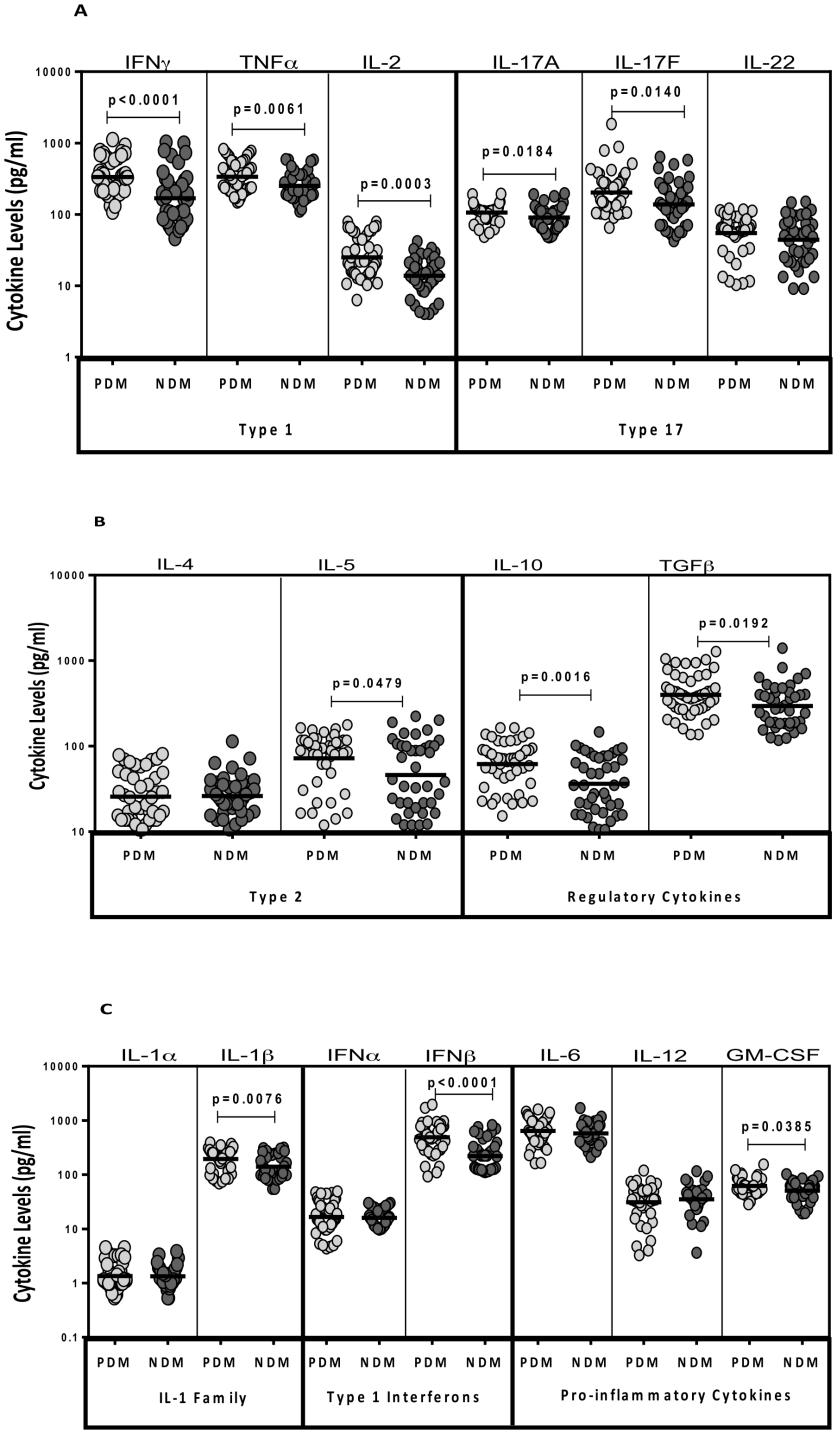
Elevated systemic levels of Type 1, Type 2 and other pro-inflammatory and regulatory cytokines in TB-PDM. The plasma levels of Type 1 (IFNγ, TNFα, IL-2); Type 17 (IL-17A, IL-17F and IL-22) cytokines (A) and Type 2 (IL-4, IL-5, IL-13); regulatory (IL-10 and TGFβ) cytokines (B) and other pro-inflammatory (IL-1α, IL-1β, IFNα, IFNβ, IL-6, IL-12 and GM-CSF) were measured by ELISA in TB-PDM (n = 48) and TB-NDM (n = 42) individuals. The data are represented as scatter plots with each circle representing a single individual (light grey – PDM and dark grey – NDM). P values were calculated using the Mann-Whitney test.

To determine the influence of PDM on Type 1 and Type 17 cytokines in active PTB, we measured the circulating levels of IFN-γ, TNFα and IL-2 as well as IL-17A, IL-17F and IL-22 in TB-PDM and TB-NDM individuals (Figure 1). As shown in Figure 1A, the systemic levels of all three Type 1 cytokines – IFNγ (Geometric Mean of 335.9 pg/ml in TB-PDM versus 169.2 pg/ml in TB-NDM), TNFα (GM of 338.4 pg/ml vs. 252.4 pg/ml) and IL-2 (GM of 25.1 pg/ml vs. 13.9 pg/ml) were significantly higher in TB-PDM compared to TB-NDM individuals. Similarly, the systemic levels of the prototypical Type 17 cytokines – IL-17A (GM of 106.3 pg/ml vs. 90.6 pg/ml) and IL-17F (GM of 202.8 pg/ml vs. 138.1 pg/ml) were also significantly higher in TB-PDM compared to TB-NDM individuals. In contrast, no significant differences in IL-22 levels were found between the two groups. Thus, TB-PDM is associated with heightened levels of Type 1 and Type 17 at the time of presentation of active PTB.

### TB-PDM is associated with increased circulating levels of Type 2 and regulatory cytokines

To determine the influence of PDM on Type 2 and regulatory cytokines in active TB, we measured the circulating levels of IL-4 and IL-5 as well as IL-10 and TGFβ in TB-PDM and TB-NDM individuals (Figure 1). As shown in Figure 1B, the systemic levels of IL-5 (GM of 72.1 pg/ml vs. 46.1 pg/ml) were significantly higher in TB-PDM compared to TB-NDM individuals. Similarly, the systemic levels of the regulatory cytokines - IL-10 (GM of 61.6 pg/ml vs. 36.3 pg/ml) and TGFβ (GM of 398.2 pg/ml vs. 295.6 pg/ml) were also significantly higher in TB-PDM compared to TB-NDM individuals. Thus, TB-PDM is not associated with a concomitant decrease of Type 2 or regulatory cytokine levels at the time of presentation with active PTB.

### TB-PDM is associated with increased circulating levels of other pro-inflammatory cytokines

To determine the influence of PDM on the IL-1 family and other pro-inflammatory cytokines as well as Type 1 IFNs in active TB, we measured circulating levels of these in TB-PDM and TB-NDM individuals (Figure 1). As shown in Figure 1C, the systemic levels of IL-1β (GM of 195 pg/ml vs. 140.1 pg/ml) and GM-CSF (GM of 61.8 pg/ml vs. 50.9 pg/ml) were significantly higher in TB-PDM compared to TB-NDM individuals. In contrast, the systemic levels of IL-1α, IL-6 and IL-12 were not significantly different between the two groups. Similarly, the levels of IFNβ (GM of 488.3 pg/ml vs. 220.5 pg/ml) but not IFNα were found be present at significantly higher levels in TB-PDM compared to TB-NDM individuals. Thus, TB-PDM is associated with heightened levels of pro-inflammatory cytokines at the time of presentation.

### TB-PDM is associated with increased TB antigen stimulated levels of pro-inflammatory cytokines

**Figure 2:**
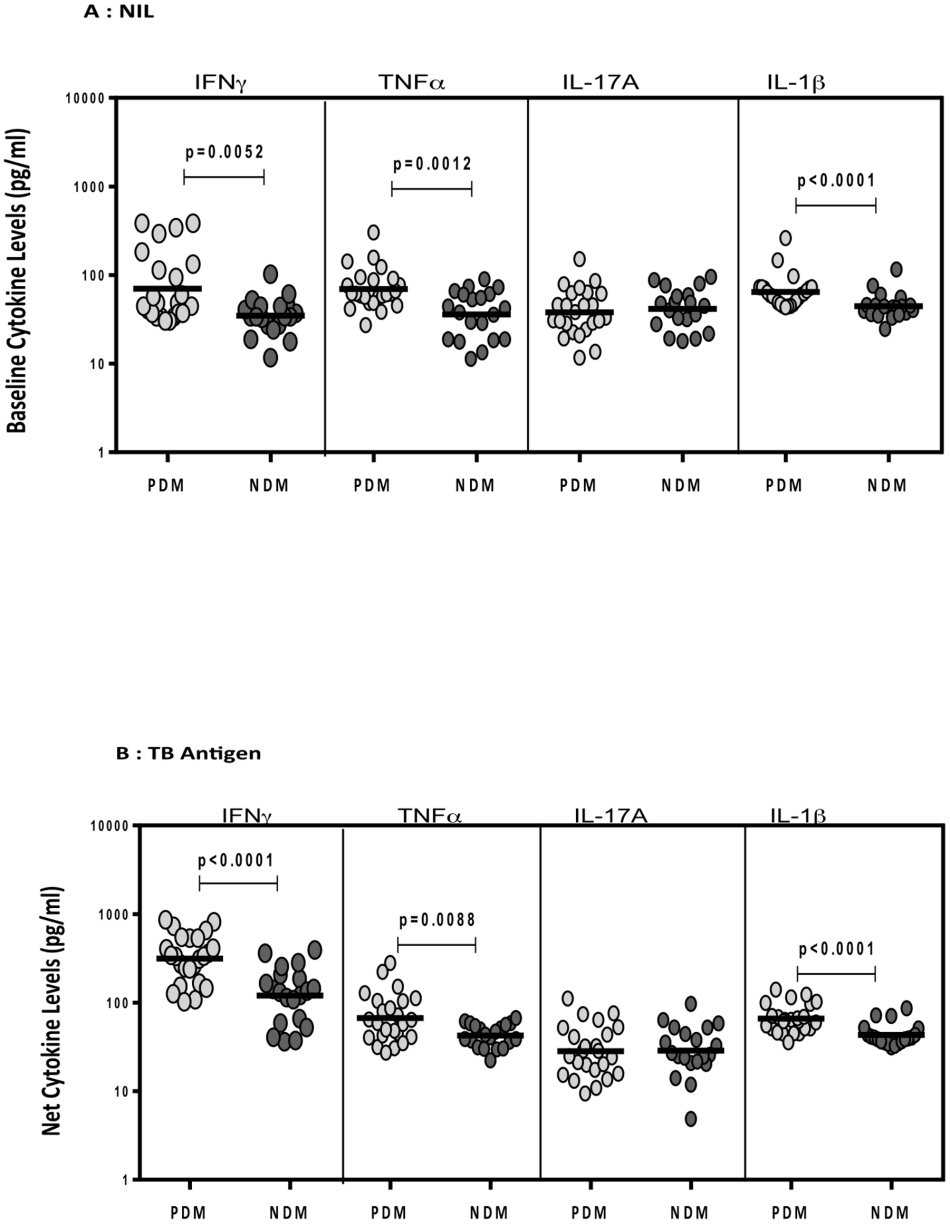
Elevated TB antigen stimulated and unstimulated levels of IFNγ, TNFα, IL-17A and IL-1β in TB-PDM. The unstimulated (A) or TB antigen stimulated (B) levels of IFNγ, TNFα, IL-17A and IL-1β were measured by ELISA in whole blood of a subset of TB-PDM (n = 22) and TB-NDM (n = 22) individuals. The data are represented as scatter plots with each circle representing a single individual (light grey – PDM and dark grey – NDM). P values were calculated using the Mann-Whitney test.

To determine the influence of PDM on TB antigen stimulated cytokine production in active PTB, we measured circulating levels of these cytokines following stimulation of whole blood with no antigen or a cocktail of TB antigens (ESAT-6, CFP-10, TB 7.7) in a subset (n = 22 each) of TB-PDM and TB-NDM individuals (Figure 2). As shown in Figure 2A, the spontaneously produced levels of the IFNγ (GM of 70.1 pg/ml vs. 34.8 pg/ml), TNFα (GM of 69.7 pg/ml vs. 35.6 pg/ml) and IL-1β (GM of 64.4 pg/ml vs. 44.5 pg/ml) were significantly higher in TB-PDM compared to TB-NDM individuals. Similarly, as shown in Figure 2B, the TB antigen stimulated levels of IFNγ (GM of 278.8 pg/ml vs. 120.1 pg/ml), TNFα (GM of 67.2 pg/ml vs. 42.5 pg/ml) and IL-1β (GM of 65.9 pg/ml vs. 43.1 pg/ml) were also significantly higher in TB-PDM compared to TB-NDM individuals. Thus, TB-PDM is associated with heightened levels of TB antigen stimulated pro-inflammatory cytokines.

### No relationship between systemic cytokines and HbA1c levels or with bacterial burdens

**Figure 3:**
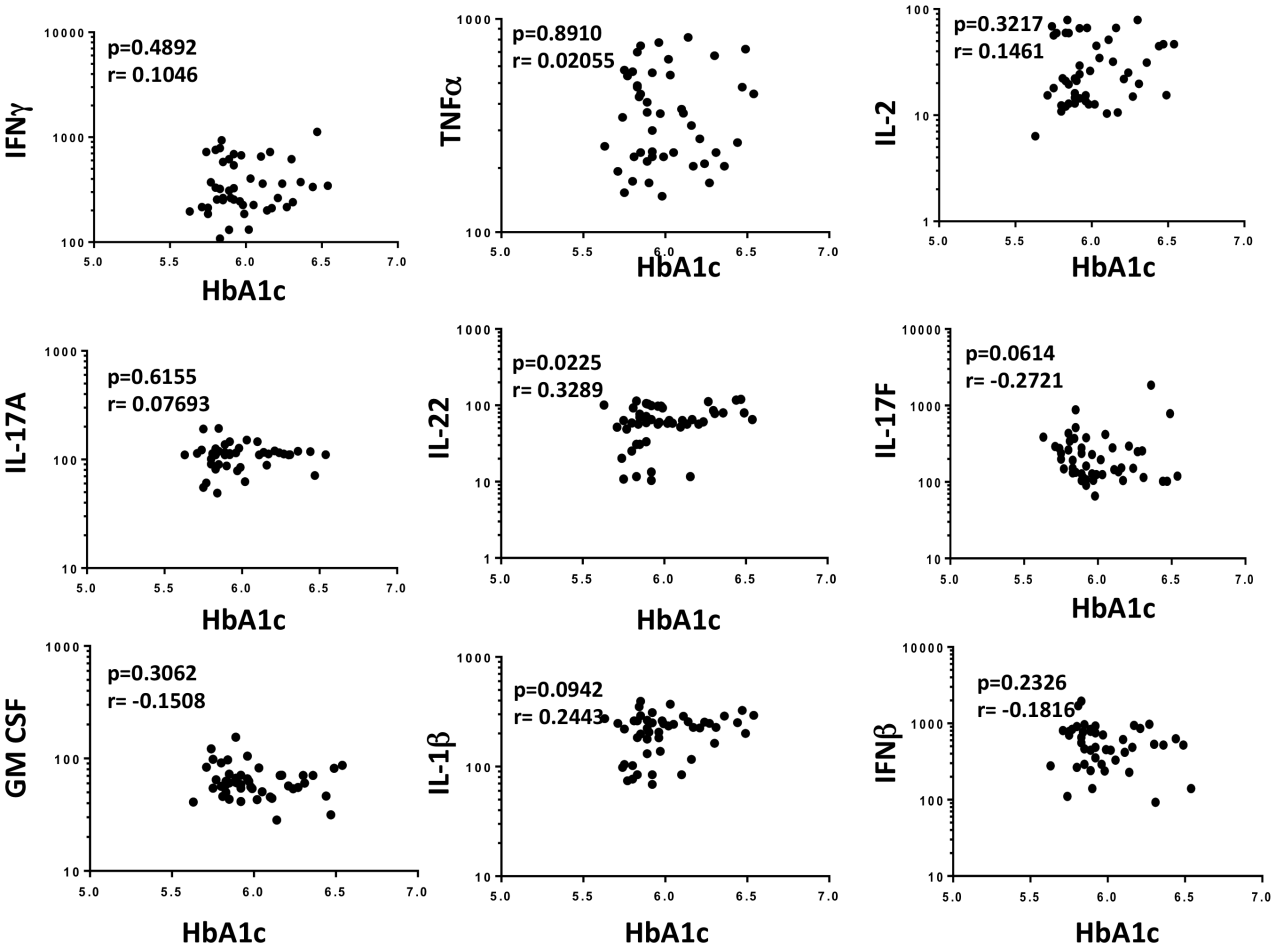
No relationship between systemic levels of cytokines and HbA1c levels in TB – PDM individuals. The relationship between the plasma levels of IFNγ, TNFα, IL-2, IL-17A, IL-17F, IL-22, GM-CSF, IL-1β and IFNβ and HbA1c levels was examined in TB - PDM (n = 48) individuals. The data are represented as scatter plots with each circle representing a single individual. P values were calculated using the Spearman rank correlation.

Since HbA1c is an accurate indicator of the level of glycemic control and increased values reflect poor control, we sought to examine the relationship between the systemic levels of Type 1, Type 17 and other pro-inflammatory cytokines with the degree of glycemic control. To this end, we assessed the association of IFNγ, TNFα, IL-2, IL-17A, IL-17F, IL-22, IL-1β, GM-CSF and IL-12 with HbA1C levels (in %) in the PDM individuals in the study. As shown in Figure 3, we observed no significant correlation between systemic cytokine levels and the HbA1c levels in TB-PDM individuals.

**Figure 4:**
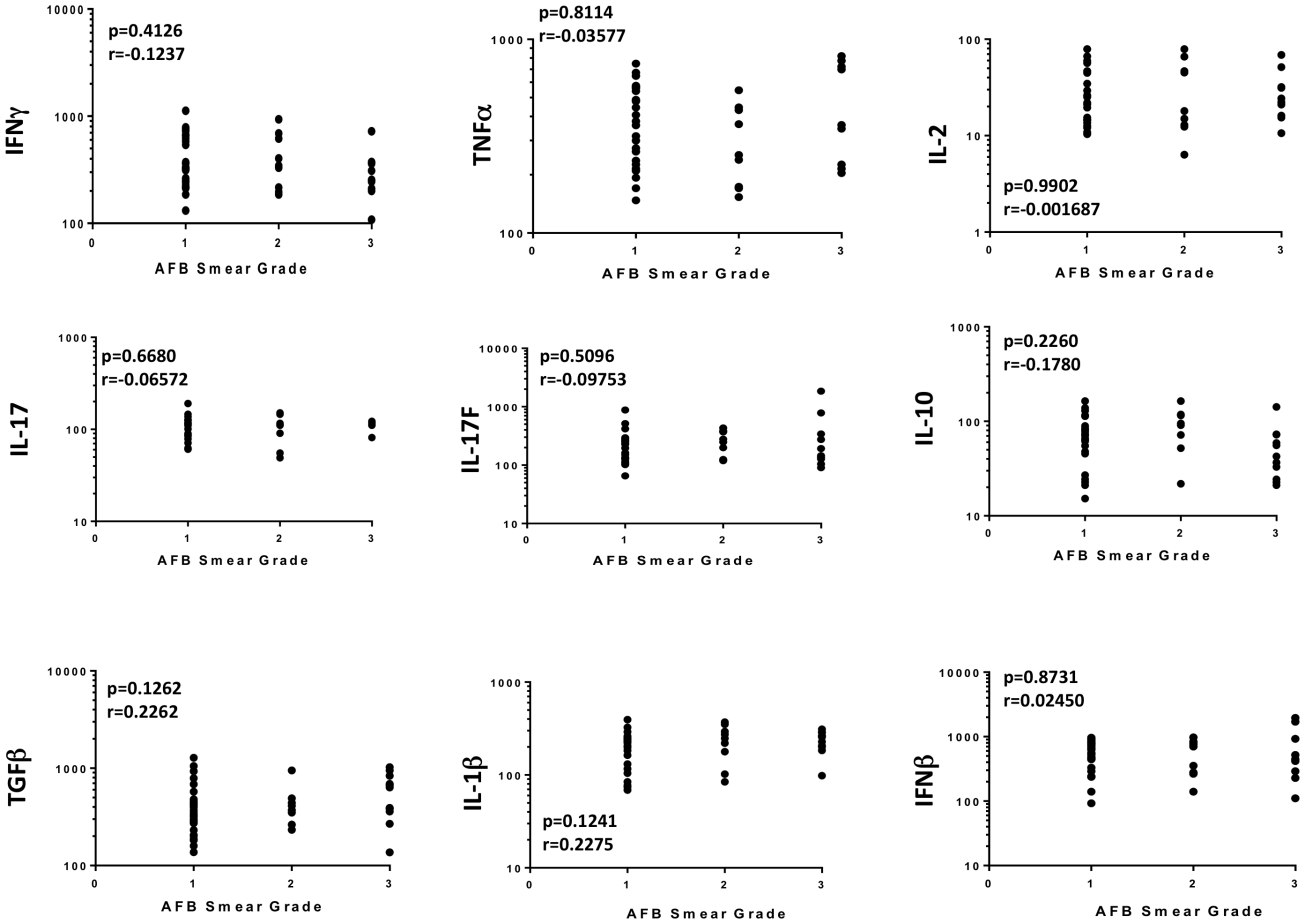
No relationship between systemic levels of cytokines and bacterial burdens in TB – PDM individuals. The relationship between the plasma levels of IFNγ, TNFα, IL-2, IL-17A, IL-17F, IL-10, IL-1β, IFNβ, TGFβ and bacterial burdens (as assessed by smear grades - 1^+^, 2^+^ and 3^+^) was examined in TB - PDM (n = 48) individuals. The data are represented as scatter plots with each circle representing a single individual. P values were calculated using the Spearman rank correlation.

Since smear grades are an accurate reflection of bacterial burdens, we also sought to examine the relationship between the systemic levels of Type 1, Type 17 and other pro-inflammatory cytokines with the bacterial burdens in the TB-PDM individuals. As shown in Figure 4, we observed no significant correlation between the circulating levels of IFNγ, TNFα, IL-2, IL-17A, IL-17F, IL-10, IL-1β, IFNβ and TGFβ.

Thus, systemic levels of cytokines in TB-PDM do not reflect poor glycemic controls or increased bacterial burdens.

## Discussion

PDM prevalence is increased in TB – it is presently unknown whether TB is causing PDM in susceptible individuals, or if PDM increases susceptibility to TB. Either possibility would have significant public health implications [12], [13]. If PDM impairs host defense and increases the risk for progression from LTBI to active TB, then the TB burden attributable to metabolic syndromes would be far greater than current estimates. It would also imply the existence of distinct susceptibility mechanisms unrelated to hyperglycemia but likely shared in PDM and type 2 DM. Alternatively, TB disease might promote insulin resistance and induce PDM as a consequence of inflammatory stress. In that scenario, TB would represent a significant factor contributing to disordered glucose metabolism with implications for bidirectional screening [14], [15]. It would then be important to learn whether this is a transient or persistent effect and whether TB can push pre-diabetics to full DM with all of its negative consequences.

Inflammation - induced inhibition of the insulin signaling pathway can lead to insulin resistance and contribute to the development of Type 2 DM [16]. Pre-diabetes and insulin resistance are associated with a chronic but subclinical inflammatory process that impairs insulin action in most tissues and could also hamper pancreatic beta-cell function [17]. The involvement of cytokines induced by this inflammation suggest an innate immune response [18]. Moreover, components of the immune system are altered in PDM, including altered levels of specific cytokines and chemokines, changes in the number and activation state of various immune cell subsets and increased apoptosis and tissue fibrosis [17]. Together, these changes suggest that inflammation participates in the pathogenesis of PDM but how these changes affect the immune response to bystander antigens or newly acquired infections remains unclear [19]. One possible mechanism is that an impaired immune response in PDM facilitates either primary infection with tuberculosis or reactivation of latent tuberculosis [20], [21].

Cytokines are known to play a major role in determining the outcome of mycobacterial infections [22]. However, unbalanced cytokine responses can also play an important role in promoting pathology in TB disease [23]. Thus, while Type 1 and Type 17 cytokines typically impart protection against TB infection [24], excess of TNFα [25] or IL-17 [26] are known to be associated with deleterious effects in TB disease. Moreover, Type 1 IFN driven gene expression signatures are considered a characteristic feature of active TB disease [27] and Type 1 IFNs are known to promote pathology in both animal models and human disease [28], [29]. Our data clearly reveal that the PDM - TB nexus is characterized by heightened circulating levels of most of the pro-inflammatory cytokines. So, contrary to their expected role in host protection, these cytokines are more likely to be associated with promotion of pathology in PTB. This is very similar to our previous findings in overt DM and PTB, where we had observed not only elevated plasma levels of pro-inflammatory cytokines but also increased TB antigen - specific Th1 and Th17 cellular and cytokine responses [6], [30].

One potential mechanism for the increase in levels of pro-inflammatory cytokines in TB-PDM individuals could be a concomitant decrease in the levels of systemic Type 2 or regulatory cytokines. However, as revealed by the increased systemic levels of IL-5, IL-10 and TGFβ, our data show that even regulatory cytokines are present at elevated levels in PDM individuals. Since Type 2 cytokines, IL-10 and TGFβ are known down modulators of inflammatory responses in TB [31], [32], this suggests that there is a compensatory mechanism in place to regulate inflammation in the setting of PDM. Another possibility is that there is a broad and possibly unrelated increase in T cell activation responses (reflected by cytokines) in PDM. Thus, PDM in the context of active PTB is characterized by a predominantly balanced network of pro - and anti - inflammatory cytokines, accounting for the lack of differences in bacterial loads between the 2 groups studied. Moreover, systemic levels of cytokines do not exhibit any significant association with bacterial burdens, suggesting that this dysregulation of cytokine levels is not solely driven by differences in bacterial burdens.

The major limitation in analyzing the immune parameters in the circulation is the non – specific nature of the analysis. We have therefore, sought to corroborate the plasma cytokine results by measuring the levels of a subset of cytokines in TB antigen stimulated whole blood supernatants. Our data on the cytokine responses following TB antigen stimulation clearly show that PDM has a profound effect and that elevation in circulating levels of Type 1 cytokines as well as IL-1β is reflected by similar elevations of in vitro measured cytokines following stimulation with mycobacterial antigens in TB-DM. Whether the heightened responsiveness observed in the TB-PDM individuals is specific to TB infection or is a general phenomenon applicable to different infections remains to be determined. While our data clearly provide descriptive data on the interaction between PDM and TB, future studies are needed to explore the mechanism governing this important interaction. Our data on the lack of differences in BMI and lipid metabolism appears to exclude these parameters as potential confounders (see Table 1). However, other metabolic mediators including free fatty acids that are known to be induced in insulin resistance could also play a role in this interaction. In addition, our data also reveal that the baseline frequencies of nTregs and CD8^+^ effector memory T cells are significantly different between the two groups, thereby making highly unlikely that significant perturbations in CD4^+^ or CD8^+^ T cell numbers are responsible for the differential cytokine profiles. The cellular sources of the cytokines produced should provide us clues to the mechanism behind this differential response. Finally our data argue that the interaction of active TB with pre-diabetes is in contrast to that of latent TB with diabetes, since in the latter scenario, impairment of cytokine secretion is the major finding [33]. Hence, pre-diabetes interaction with latent TB potentially enhances susceptibility to TB disease by compromising the production of protective cytokines, while pre-diabetes interaction with active TB potentially promotes pathology by enhancing the production of cytokines.

Our study provides important insights into the influence of pre-diabetes on the pathogenesis of TB disease. Our data also suggest that this interaction might also have an effect on TB disease potentially shifting the balance of metabolic regulation from pre-diabetes to overt diabetes. These data underlie the need to assess more systematically – using epidemiological, clinical and immunological studies – the interaction between pre-diabetes and tuberculosis and suggests that intervention that targets important innate and adaptive responses may provide new clues to pursue immunological alternatives as therapeutics for dealing with the global menace of coincident diabetes and TB.

## Supporting Information

Figure S1 **Gating strategy for estimating frequencies of CD4^+^ and CD8^+^ naïve, central memory and effector memory T cells and natural regulatory T cells.** (A) A representative flow cytometry plot showing the gating strategy for estimation of naïve, central memory and effector memory cells from CD4^+^ and CD8^+^ T cells. Naïve cells were classified as CD45RA^+^ CCR7^+^; effector memory cells as CD45RA- CCR7-; and central memory cells as CD45RA- CCR7^+^. (B) A representative flow cytometry plot showing the gating strategy for estimation of nTregs from CD4^+^ T cells. Natural T regulatory T cells (nTregs) were classified as CD4^+^, CD25^+^, Foxp3^+^, CD127dim.
(TIFF)
