# Supplementary figures and images for: Coincident Pre-Diabetes Is Associated with Dysregulated Cytokine Responses in Pulmonary Tuberculosis
Source: PLoS One. 2014 Nov 13;9(11):e112108. doi: 10.1371/journal.pone.0112108 (PMC4230980; doi:10.1371/journal.pone.0112108)

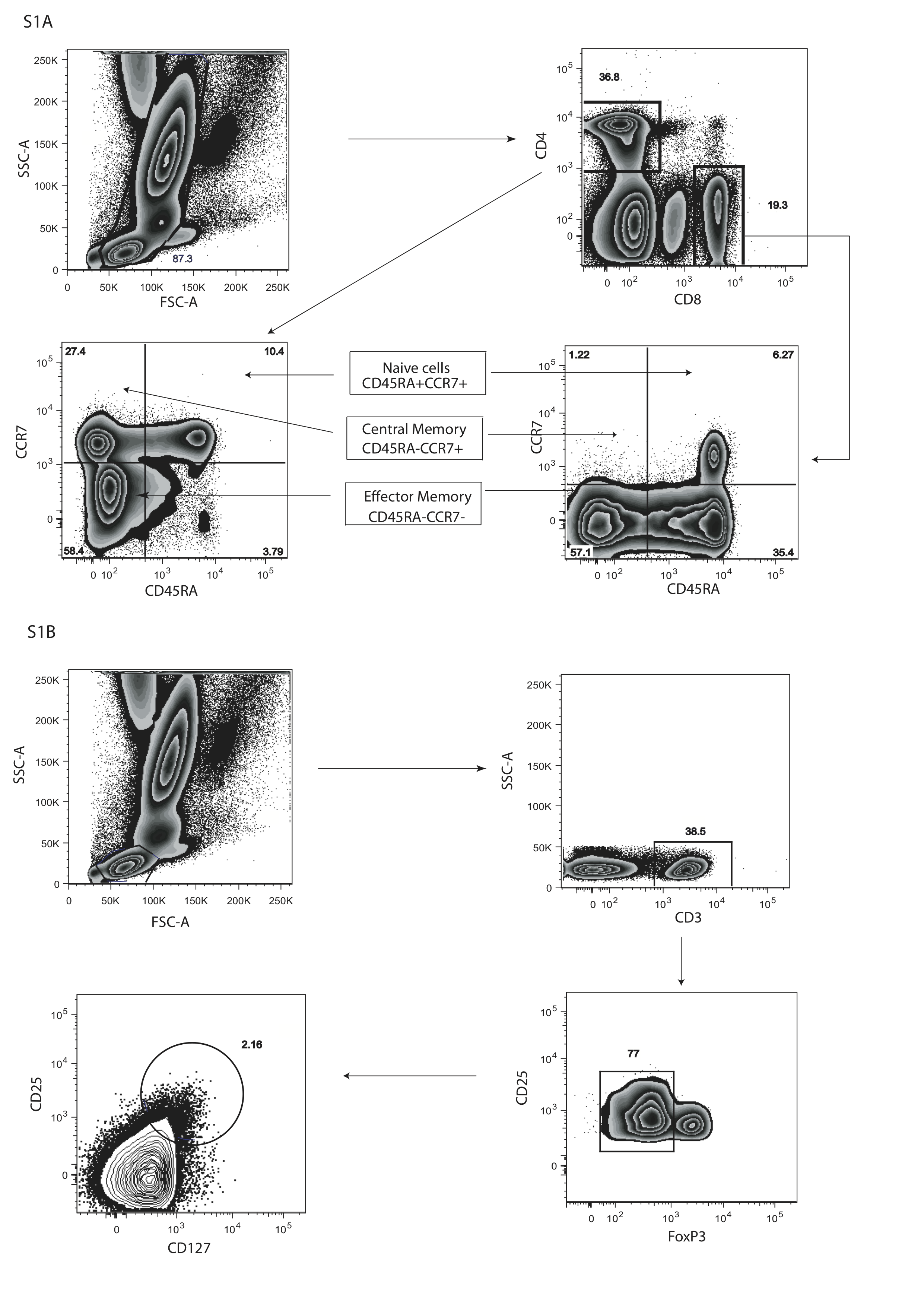

Supplement: Figure S1 — Gating strategy for estimating frequencies of CD4+ and CD8+ naïve, central memory and effector memory T cells and natural regulatory T cells. (A) A representative flow cytometry plot showing the gating strategy for estimation of naïve, central memory and effector memory cells from CD4+ and CD8+ T cells. Naïve cells were classified as CD45RA+ CCR7+; effector memory cells as CD45RA- CCR7-; and central memory cells as CD45RA- CCR7+. (B) A representative flow cytometry plot showing the gating strategy for estimation of nTregs from CD4+ T cells. Natural T regulatory T cells (nTregs) were classified as CD4+, CD25+, Foxp3+, CD127dim. (TIFF) [file pone.0112108.s001.tiff]
